# Hygiene of Medical Devices and Minimum Inhibitory Concentrations for Alcohol-Based and QAC Disinfectants among Isolates from Physical Therapy Departments

**DOI:** 10.3390/ijerph192214690

**Published:** 2022-11-09

**Authors:** Urška Rozman, Darja Duh, Mojca Cimerman, Sonja Šostar Turk

**Affiliations:** 1 Faculty of Health Sciences, University of Maribor, Žitna ulica 15, 2000 Maribor, Slovenia; 2 Chemicals Office of the Republic of Slovenia, Ajdovščina 4, 1000 Ljubljana, Slovenia; 3 National Laboratory of Health, Environment and Food, Prvomajska ulica 1, 2000 Maribor, Slovenia

**Keywords:** surfaces hygiene, disinfectants, MIC, healthcare institution

## Abstract

Disinfectants are used intensively to control and prevent healthcare-associated infections. With continuous use and exposure to disinfectants, bacteria may develop reduced susceptibility. The study aimed to check the hygiene of devices in the physiotherapy department. For isolated bacterial strains, we aimed to determine the minimum inhibitory concentration of five different disinfectant wipe products currently in use. Microbiological environmental sampling in four various institutions in four different cities from two counties was performed, followed by CFU calculation and identification using matrix-assisted laser desorption and ionization with time-of-flight analyzer mass spectrometry (MALDI-TOF). The sampling was performed on three different occasions: before patient use, after patient use, and after disinfection. The susceptibility of isolates to three different alcohol-based and three different quaternary ammonium compounds (QAC) disinfectant wipes was examined by determining the minimal inhibitory concentrations (MIC). We identified 27 different bacterial species from 11 different genera. Gram-positive bacteria predominated. The most abundant genera were *Staphylococcus*, *Micrococcus*, and *Bacillus*. The average MIC values of alcohol-based disinfectants range between 66.61 and 148.82 g/L, and those of QAC-based disinfectants range between 2.4 and 3.5 mg/L. Distinctive strains with four-fold increases in MIC values, compared to average values, were identified. The widespread use of disinfectants can induce a reduction in the susceptibility of bacteria against disinfectants and affect the increase in the proportion of antibiotic-resistant bacteria. Therefore, it is urgent to define clear criteria for defining a microorganism as resistant to disinfectants by setting epidemiological cut-off (ECOFF) values and standardizing protocols for testing the resistance of microorganisms against disinfectants.

## 1. Introduction

Physical therapy equipment and tools are a group of medical devices and accessories that come in direct contact with the patient’s skin and may therefore facilitate the indirect transmission of microorganisms among patients [1,2,3,4]. To control and prevent the spread of microorganisms that can cause healthcare-associated infections, hospitals and other healthcare facilities use disinfectants intensively [5,6]. The global biocidal market and consumption of disinfectants are increasing, and the biocides market size is estimated to be USD 11.3 billion in 2021 and projected to reach USD 13.6 billion by 2026. On top of that, the global COVID-19 pandemic caused an even higher demand and consumption of disinfectants for household and personal care [7]. With continuous use and overexposure to disinfectants, the development of reduced susceptibility of bacteria to equal doses of disinfectants [8] in clinical, domestic, or industrial settings may be enhanced [9]. Due to the increased use of biocidal products worldwide, especially those that are commonly used, it is not surprising that resistance develops. Biocidal active compounds that are released into the environment constantly produce selective pressure for resistant mechanisms [10]. Therefore, more attention should be paid to biocide products, disinfectants for human personal hygiene, and surface/material disinfectants in a healthcare environment in particular [11].

Bacterial survival after exposure to biocides has been recognized for decades, yet the topic has received little attention as opposed to antibiotics [12]. While antibiotic resistance is a well-recognized and researched topic in the scientific and medical communities [13,14,15,16], less attention is paid to the possible cross-resistance and/or co-resistance mechanisms in the case of disinfectants [5,17,18,19]. Although biocide resistance development is unlikely [9,20,21], various inside and outside cellular mechanisms can lead to decreased susceptibility of microorganisms against disinfectants [22].

The study aimed to check the hygiene of devices in the physiotherapy department intended for multiple uses and to examine the microbial population on these surfaces on three different occasions: before patient use, after patient use, and after disinfection. For isolated bacterial strains, we aimed to determine the minimum inhibitory concentration of six different disinfectant wipe products currently used in order to detect potentially reduced susceptibility of bacterial strains. Minimum inhibitory concentrations (MICs) are defined as the lowest concentration of an antimicrobial that will inhibit the visible growth of a microorganism after overnight incubation.

## 2. Materials and Methods

Sampling and isolation: the surfaces of selected reusable devices in physiotherapy rooms and devices and equipment in other rooms in participating healthcare facilities were sampled using pre-moistened swabs with SRK^®^ Neutralizing Transport Medium (Copan). Each selected device was sampled on an approximately 25 cm^2^ area on three different occasions: before use, after use, and after disinfection. The samples were collected over multiple days, whereas samples from one institution were collected on the same day. The list of sampled devices and equipment across different facilities and institutions is listed in Supplementary Material File S1.

The concentration of the microbial population and the isolation of pure cultures: to determine the concentration of the microbial population, viable plate counting on TSA (Tryptic Soy Agar) was used, along with the preparation of serial ten-fold dilutions of the original sample. After incubation, colonies were counted, and the CFU/mL was calculated.

Bacteria were isolated from 500 µL of the original sample that was plated on Tryptic Soy Agar plates and incubated for 24 h at 37 °C. Pure cultures were obtained from different colonies, selected based on differing morphology, which were once again transferred to TSA and incubated for 24 h at 37 °C.

Species identification: bacteria were identified at the species level using matrix-assisted laser desorption and ionization with time-of-flight analyzer mass spectrometry (MALDI-TOF) with MALDI Biotyper Smart (Bruker Daltonics, Billerica, MA, USA), and samples were analyzed immediately. Data acquisition and processing were performed with the MBT Compass (version 4.1) (Bruker Daltonics GmbH, Billerica, MA, USA, ZDA) and FlexControl (version 3.4) (Bruker Daltonics GmbH, Billerica, MA, USA, ZDA) software. The data acquisition mass range was m/z 4.000–17.000 Da.

Biocides and disinfectants: the biocidal wipe products used in the sampled institutions were in the form of wipes, where the disinfectant was ready for use and no pre-treatment or dilution of the products was required. All isolates were tested to determine the MIC for five disinfectants:

(i) Incidin Liquid (Ecolab, Maribor, Slovenia): 35 g propan-2-ol and 25 g propan-1-ol in 100 g solution;
(ii) Sani-Cloth 70% (PDI): 70 mL isopropyl alcohol in 100 mL solution;
(iii) Descosept Sensitive Wipes (Dr. Schumacher): 45 g ethanol in 100 g solution;
(iv) Mikrozid Sensitive Liquid (Schülke & Mayr, Norderstedt, Germany): 0.25 g alkyl dimethylbenzyl ammonium chloride, 0.25 g didecyldimethylammonium chloride, and 0.25 g alkyl ethylbenzylammonium chloride in 100 g solution;
(v) L + R Surface Disinfect Universal (Lohmann & Rauscher, Rengsdorf, Germany): 0.4 g alkyl dimethylbenzyl ammonium chloride and 0.4 g didecyldimethyl ammonium chloride in 100 g solution.

Minimum Inhibitory Concentration (MIC) Determination: the susceptibility of isolates to alcohol-based and QAC disinfectants used in participating institutions was verified by the determination of the minimum inhibitory concentration (MIC). To prepare an inoculum suspension (2 mL), we adjusted the turbidity to the 0.5 McFarland standard, which corresponds to approximately 1 − 2 × 10^8^ CFU/mL [23]. Then, 25 μL of this bacterial suspension was transferred to 5 mL of TSB, resulting in approximately 2.5 − 5 × 10^5^ CFU/mL in the well. 

A total of 100 μL of Mueller Hinton broth was included in all wells of a sterile 96-well microtiter plate. In the first row (row A), 200 μL of a working concentration of biocide solution was added for alcohol-based disinfectants. 

For Quaternary Ammonium Compounds (QAC) disinfectants, the biocide solution was six times diluted for the first row since the expected MICs were lower. Then, 100 μL of biocide solution from row A was added to row B, and the exact steps were repeated until the lowest test concentration in row G was reached. After mixing, 100 μL of solution from row G was withdrawn. Row H contained no biocide and served as growth control. Finally, all wells of the microtiter plate were inoculated with 100 μL of the bacterial suspension [24,25,26]. These steps were followed for each sample from every piece of equipment at all four facilities at all three time points (before-patent, after-patient, and after disinfection).

## 3. Results

### 3.1. Hygiene of Devices in the Physiotherapy Department

The surfaces of selected reusable devices in different departments were sampled (Supplementary Material File S1). Three different types of healthcare facilities were involved: the university clinical center (institution A), nursery home (institution B), private rehabilitation clinic (institution C), and university rehabilitation institute (D). To obtain the most realistic results possible, sampling took place during therapy. Therefore, samples could not be taken on some devices at certain times (marked as/in the Supplementary Material File S1).

**Figure 1:**
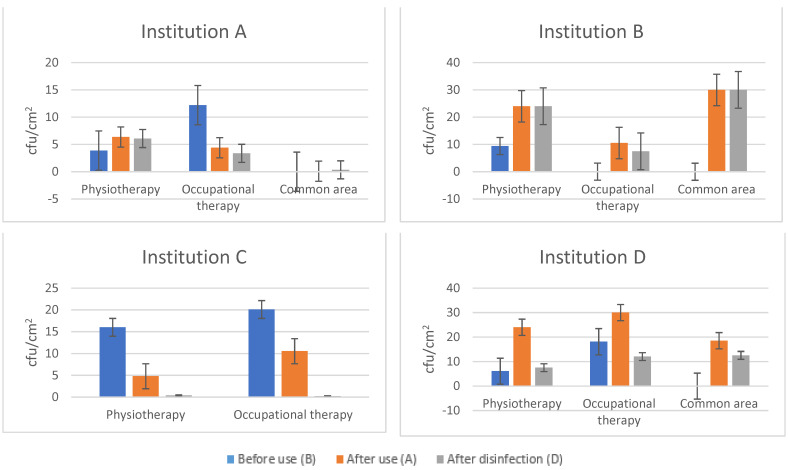
Average CFU/cm^2^ values across facilities sampled on three different occasions: before use, after use, and after disinfection.

In Figure 1 and Table 1, the data about average CFU/cm^2^ values across equipment and facilities are presented. In all four institutions, the average value of CFU/cm^2^ after disinfection is lower than the value of CFU/cm^2^ after use. To evaluate our assumption that the values after disinfection were lower than after use, the one-sided paired *t*-test was performed. Only the Institution D results indicate a statistical difference (t = 3.129; *p* = 0.005), whereas in the other three institutions, there is no statistical difference between values after use and after disinfection (Institution A, t = 0.204, *p* = 0.421; Institution B, t = 0.330, *p* = 0.374; and Institution C, t = 1.262, *p* = 0.148). Limited sample sizes and real-world data samples precluded a more complex analysis.

To evaluate the disinfection efficacy of alcohol and QAC wipe products, the after-disinfection CFUs were compared to the after-patient interaction CFUs, calculating the log reduction. Only in Institution C was a value over 1 log reduction observed (Figure 1). Limited sample sizes and real-world data samples precluded a more complex analysis.

**Figure 2:**
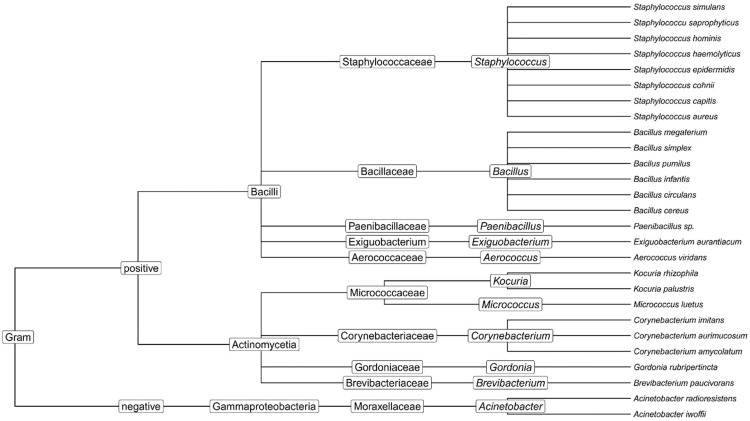
Dendrogram of the cluster analysis results for identified species found across institutions and facilities.

With the use of MALDI-TOF technology, we were able to identify 27 different bacterial species that belonged to 11 different genera at all four institutions. Gram-positive bacteria predominated, with 25 species identified as Gram-positive bacteria and two as Gram-negative bacteria. (Figure 2). 

### 3.2. Minimum Inhibitory Concentrations (MIC)

**Figure 3:**
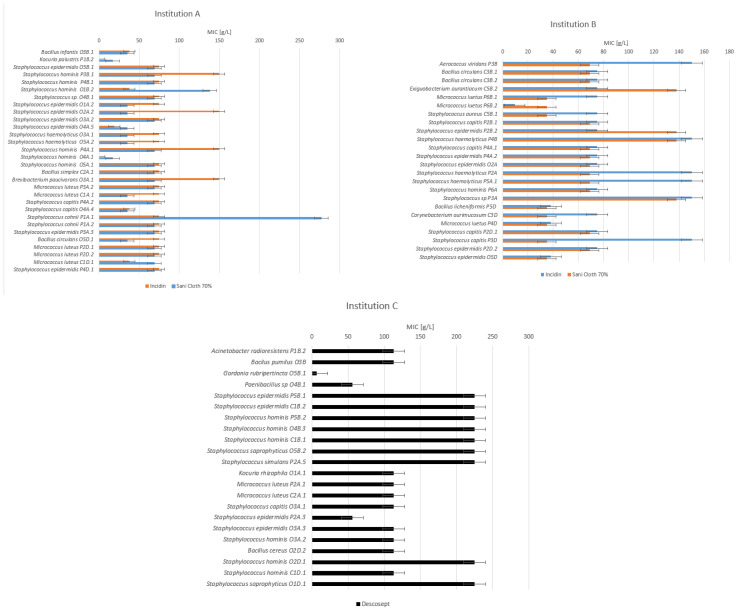
MIC values [g/L] for alcohol-based disinfectants Incidin, Sani-Cloth 70%, and Descosept for isolates in institutions (A), (B), and (C). Legend: P—physiotherapy; O—occupational therapy; C—common area; B—before use; A—after use; and D—after disinfection. The first number is the number of the sampled device (Supplementary Material File S1); the second number is the serial number of the isolate.

**Figure 4:**
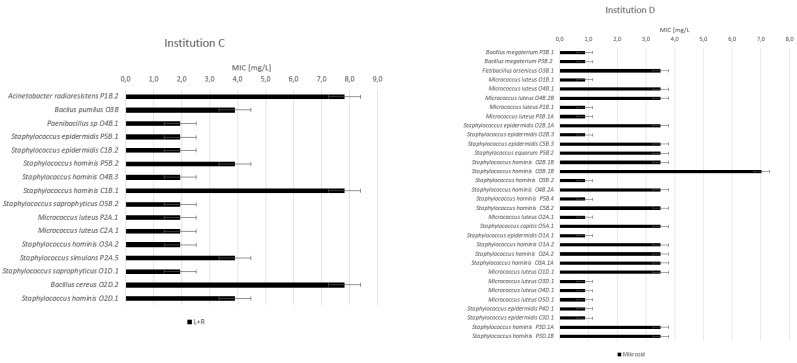
MIC values [mg/L] for QAC-based disinfectants L + R and Mikrozid for isolates in institutions (C) and (D). Legend: P—physiotherapy; O—occupational therapy; C—common area; B—before use; A—after use; and D—after disinfection. The first number is the number of the sampled device (Supplementary Material File S1), and the second number is the serial number of the isolate.

To determine the changed or reduced susceptibility of isolates against selected disinfectants that are in use in the participating healthcare institutions, the MIC method was used. The following results provide information about MIC values for disinfectants Incidin Liquid (Ecolab), Sani-Cloth 70% (PDI), Descosept Sensitive Wipes (Dr. Schumacher), Clinell Universal Wipes (GAMA Healthcare, Hemel Hempstead, UK), Mikrozid Sensitive Liquid (Schülke & Mayr), and L + R Surface Disinfect Universal (Lohmann & Rauscher) (Figure 3 and Figure 4).

The average MIC values of the alcohol-based disinfectants Incidin and Sani-Cloth in both institutions at all sampling occasions are 82.10 g/L and 66.61 g/L, respectively. Distinctive strains were noticed, for which the MIC values for Incidin and Sani-Cloth reached 70% at 150 g/L. In particular, a high MIC was observed for Sani-Cloth 70% concerning *Staphylococcuss cohnii* in institution A at the physiotherapy department on the therapeutic pillow after use. For the alcohol-based disinfectant Descosept, the average MIC value at all sampling occasions is 148.82 g/L, but for the majority of *Staphylococcus* strains, those are at 225 g/L.

For the QAC based disinfectants L + R and Mikrozid, the average MIC values in both institutions at all sampling occasions are 3.5 and 2.4 mg/L, respectively. The distinctive strains showing MIC values for L + R disinfectant at 7.8 mg/L were:

- *Acinetobacter radioresistens* in institution C at the physiotherapy department on the therapeutic pillow before use;
- *Bacillus cereus* in institution C at the occupational therapy department on an LED monitor after disinfection;
- *Staphylococcus hominis* in institution C from the common area on the keyboard before use.

For the disinfectant Mikrozid in institution D, the highest observed MIC was at 7.0 mg/L for *Staphylococcus hominis* from the occupational therapy department on a hand bike before use.

In Table 2, the average MIC values for all five tested disinfectants across facilities at different sampling occasions are presented. 

The average MIC values for tested disinfectants in institutions A, B, and D are always the lowest at sampling occasions after disinfection. According to one-sided paired *t*-test analysis, the statistically significant differences between sampling after use and after disinfection are observed for the disinfectant Sani-Cloth 70% in Institutions A (*p* = 0.035) and B (*p* = 0.020), and for the disinfectant Incidin in Institution B (*p* = 0.031). 

## 4. Discussion

Equipment and surfaces in hospitals and health facilities are important routes of transmission of microorganisms between patients, visitors, and healthcare professionals, so thorough cleaning, disinfection, or sterilization of reusable surfaces and objects is essential to control and prevent the spread of nosocomial infections [6,27,28]. In this research, different multiple-use objects were sampled in four different institutions in four different cities from two counties on three different occasions. By sampling the devices “before use”, we can determine the microbial population on the devices before coming into contact with the following user. When comparing the concentrations from the “before use” step to the sampling step “after use”, we get information on the microorganisms that patients transfer to devices during the therapeutical procedure. The final sampling step, “after disinfection”, provides information on the correct use of disinfectants, the effectiveness of disinfection, and the potential microorganisms that remain on the devices after disinfection. The log reduction values between “after use” and “after disinfection” values across institutions ranged between 0.028 and 1.512 log CFU/cm^2^, which is much lower than prescribed in the Guidance on the Biocidal Products Regulation, where a value of 4–5 log reduction is required before a product will be assessed to be sufficiently effective [29]. It is a worrying fact that some devices are contaminated before use, and it is particularly worrisome that they remain contaminated even after disinfection. Similar results were also observed in other studies, where the cleanliness of the devices deviated from the prescribed standards and a large part of the devices were improperly cleaned [30,31]. The most frequently encountered species on multiple-use objects regarding MALDI-TOF identification were *Staphylococcus*, *Micrococcus*, and *Bacillus,* which is consistent with the results of Afle et al. [31].

The disinfection efficiency can be attributed to various factors, such as choosing the right type and concentration of disinfectant, the time of interaction, the method of application, and the strength and direction of wiping. Every disinfectant is accompanied by instructions for use and storage, as this alone has a significant impact on the effectiveness of disinfection. When not used as directed, the role of a disinfectant can change quickly, and the disinfectant itself can become a source of infection [32]. Another important factor affecting proper hygiene is suitably educated staff equipped with instructions and training for effective disinfection [33,34]. 

Studies show that, through continuous use and exposure to disinfectants, bacteria may develop a reduced sensitivity to the same doses of disinfectant due to adaptive mechanisms [8]. The main mechanisms of antimicrobial resistance are limiting the uptake of a drug, modification of a drug target, inactivation of a drug, and active efflux of a drug. These mechanisms may be native to the microorganisms or acquired from other microorganisms [35]. The mode of action of disinfectants can be unspecific, targeting different processes or sites in bacterial cells [9,20,21], where the emergence of resistance can be promoted with mutation or amplification of an endogenous chromosomal gene, acquiring resistant determinants on plasmids, transposons, integrons, changes in cell envelope permeability, increased efflux pump expression, or specific mechanisms of phenotypic traits [36,37,38]. Decreased sensitivity and resistance against disinfectants have been documented primarily against biocidal products with less reactive compounds such as quaternary ammonium compounds, biguanides, and phenols [39] and specifically against disinfectants such as: benzalkonium chloride (BAC), quaternary ammonium compound (QAC), triclosan (TCS), polychloro phenoxy phenol, chlorhexidine (CHX) (Cieplik et al., 2019), eugenol, thymol, trichlorocarbanalide (TCC), didecyl dimethyl ammonium, chloride (DDAC), C10-16-alkyldimethyl [40], tetracycline, and chloramphenicol [41]. Two types of disinfectants based on the chemistry of active substances were tested in the present study, i.e., alcohol-based and QAC-based disinfectants. Bacterial adaptation mechanisms for alcohol-based disinfectants include horizontal gene transfer, transformation, and transduction, as well as core genome mutations in the chromosome nucleotide position on the rpoB gene subunit of RNA polymerase [42,43]. For the QAC-based disinfectants, the adaptation mechanisms include downregulation of membrane porins, overexpression or modification of efflux pumps (Mrdl, EmrE, and MdfA) with mutations of the Mex system, horizontal gene transfer of transposon elements (Tn6188) and stress factors, and biodegradation by dealkylation [44,45].

To determine resistance or decreased susceptibility against disinfectants (and antibiotics), the minimum inhibitory concentration (MIC) is widely used. The ecological concept of antibiotic resistance, as defined by the European Committee on Antimicrobial Susceptibility (EUCAST), states that “a microorganism is defined as a wild type that has no acquired and mutational mechanisms of resistance against the pathogen”. Any isolate with a MIC above the epidemiological cut-off value (ECOFF), which is the upper limit of the normal distribution of the MIC for a given antimicrobial agent and a particular species, is considered resistant [46,47]. In the case of studying biocide resistance, however, no ECOFF limits have been set so far, and there are no clear criteria to determine whether a microbe is susceptible to the biocide or not. Data on bacterial resistance to biocides is often difficult to interpret and compare due to the absence of clear criteria for defining a microorganism as resistant to disinfectants and the lack of standardized tests for examining in vitro susceptibility to disinfectants [48,49]. Depending on the publications, the bacteria is said to have “adapted” to the active substance when the MIC of the biocide is at least four times higher than the initial MIC [50].

Therefore, we can use the average MIC values obtained from individual laboratory studies conducted under relatively similar conditions. The observed relevant increase in the MIC value can indicate decreased susceptibility or even resistance. While reduced susceptibility is interpreted as an elevated MIC value or increased diameter zone in the disk diffusion assay, resistance is understood as inhibition of bacterial growth. Given the likelihood that resistant strains are selected from strains with pre-existing reduced susceptibility, this relationship may reflect the presence of distinct selective pressures responsible for each population [51,52,53,54,55]. When interpreting the results, the in-use concentration of the disinfectants used must be considered since the in-use concentration is normally higher than the actual measured MIC values. In this case, we cannot talk about the resistance but only about the decreased susceptibility [46].

In the present study, for alcohol-based disinfectants, the MIC values for the majority of isolates range between 9.4 and 150 g/L, which is comparable to other studies [56,57,58]. There was only one outstanding strain, *Staphylococcuss cohnii,* in institution A at the physiotherapy department on the therapeutic pillow after use, where the MIC value was four-fold higher than the average value of other *Staphylococcus* strains, which can indicate reduced susceptibility [50]. MIC values for other tested strains range in approximately the same range, which also supports what has already been established that alcohol-based sanitizers are not known to induce resistance in bacteria, allowing for their widespread general application [59]. For QAC disinfectants, the MIC values of isolates are in the range between 0.9 and 3.9 mg/L, which does not indicate reduced susceptibility since those values are comparable to other studies [46,60,61,62]. We identified four different strains where the MIC values were four-fold higher than average values, possibly indicating reduced susceptibility [50]. Despite this finding, monitoring of resistance against QAC-based disinfectants is important since the use of quaternary ammonium compounds (QACs) may be a potential key driver in the emergence of antimicrobial resistance [49]. Most QAC formulations do not require rinsing with water after application; thus, contact between bacteria and QACs may be prolonged [63]. Long-term exposure to a QAC with low chemical reactivity that does not rapidly neutralize might expose microbial communities to sub-inhibitory concentrations. This could favor the survival of less susceptible microbial strains [64].

It is hypothesized that the widespread use of disinfectants affects the increase in the proportion of antibiotic-resistant bacteria [6]. Bacterial resistance to commonly used disinfectants, as well as the induction of cross-resistance to antibiotics, has already been demonstrated [5,19]. If the resistance and frequency of mutations would increase and develop against many commonly used disinfectants in clinical and industrial settings, this alone could burden global public health [65]. 

## 5. Conclusions

The 2016 Report on Antimicrobial Resistance predicts a worrying scenario, as the mortality attributable to microbial resistance is expected to increase globally from the current 700,000 to 10 million deaths annually by 2050 [65]. Research in the local clinical setting is urgently needed to contribute new knowledge about the link between disinfectant use and bacterial disinfectant resistance and its association with antibiotic resistance. Given the widespread use of multiple resistant bacteria against antibiotics and the potential for increased resistance against disinfectants rising in the community, the prudent use of available and still effective antimicrobial agents is needed. The risks and benefits of using disinfectants in healthcare facilities need to be weighed in order to identify and determine additional precautions for the development and use of disinfectants. Regular monitoring of bacterial susceptibility to disinfectants would be sensible, thereby preventing the spread of bacterial resistance against disinfectants and antibiotics [66]. It would also be urgently needed to define clear criteria for defining a microorganism as resistant to disinfectants by setting ECOFF values and standardizing protocols for testing the resistance of microorganisms against disinfectants.

## Figures and Tables

**Table 1:** Average CFU/cm^2^ values across institutions, sampled on three different occasions: before use, after use, after disinfection, and after log reduction (A/D).

	Before Use (B)	After Use (A)	After Disinfection (D)	Log Reduction (A/D)
Institution A	8.04 ± 9.38	4.50 ± 6.54	4.00 ± 6.64	0.051 (11.11%)
Institution B	5.16 ± 9.62	19.21 ± 14.35	18.00 ± 15.49	0.028 (6.30%)
Institution C	18.05 ± 14.18	9.10 ±14.08	0.28 ± 0.19	1.512 (96.92%)
Institution D	12.11 ± 14.16	25.18 ± 9.56	10.52 ± 14.52	0.379 (58.22%)

**Table 2:** Average MIC values for all five tested disinfectants across facilities at different sampling occasions.

	Incidin [g/L]	Sani-Cloth 70% [g/L]	Descosept [g/L]	L + R [mg/L]	Mikrozid [mg/L]
Institution A					
Before use	75.20 ± 45.72	66.17 ± 41.41			
After use	82.33 ± 34.65	64.68 ± 54.62			
After disinfection	67.60 ± 16.55	62.20 ± 15.21			
Institution B					
Before use	83.44 ± 40.61	79.50 ± 43.01			
After use	107.14 ± 40.09	78.86 ± 26.08			
After disinfection	69.86 ± 39.89	44.71 ± 16.59			
Institution C					
Before use			163.90 ± 84.22	3.69 ± 2.48	
After use			119.88 ± 46.93	2.44 ± 0.98	
After disinfection			169.00 ± 64.66	4.55 ± 2.98	
Institution D					
Before use					2.54 ± 1.73
After use					2.63 ± 1.36
After disinfection					1.87 ± 1.36

## Data Availability

Not applicable.

## Supplementary Materials

The following supporting information can be downloaded at: https://www.mdpi.com/article/10.3390/ijerph192214690/s1, File S1: Concentration of microorganisms (cfu/mL) on reusable devices.
