# Supplementary material for: Hygiene of Medical Devices and Minimum Inhibitory Concentrations for Alcohol-Based and QAC Disinfectants among Isolates from Physical Therapy Departments
Source: Int J Environ Res Public Health. 2022 Nov 9;19(22):14690. doi: 10.3390/ijerph192214690 (PMC9691081; doi:10.3390/ijerph192214690)
Supplement: Supplementary file 1 [file ijerph-19-14690-s001.zip › ijerph-1997664-supplementary.pdf]

Supplementary Material File S1. Concentration of microorganisms (cfu/mL) on reusable devices.

	Before use (B)	After use (U)	After disinfection (D)
		[cfu/cm²]	
**INSTITUTION A**			
Physiotherapy			
therapeutic pillow P1	0,9	15,6	0
stationary bike P2	1	0	0,4
therapeutic bars P3	15	15	15
stilts P4	1,4	0,3	15
stimulation electrodes P5	1,1	0,9	0
Occupational therapy			
wooden accessorise 1 O1	30	0,2	0
wooden accessorise 2 O2	15	3	0,5
hand bike O3	15	3,2	15
therapeutic ball O4	0,9	0,6	0,1
work desk O5	0,1	1,5	1,3
Common area			
key board C1	/	0,1	0,5
coffe machine C2	/	0,1	0,2
**INSTITUTION B**			
Physiotherapy			
therapeutic pillow P1	0	0	0
stationary bike P2	30	30	30
therapeutic bars P3	2,1	30	30
stilts P4	15	30	30
stimulation electrodes P5	0	30	30
Occupational therapy			
jigsaw puzzle O1	0	0	0
wooden accessorise O2	0	0,1	0
wooden ladder O3	0,2	30	0
work desk O4	0	12	30
Common area			
keyboard C1	/	30	30
coffee machine C2	/	/	30
**INSTITUTION C**			
Physiotherapy			
therapeutic pillow P1	2	/	/
seat cushion P2	/	4,8	0,4
stimulation electrodes P3	30	/	/
OCCUPATIONAL THERAPY			
plastic accessorise O1	/	30	0,4
LED monitor O2	/	0	0,3
hand bike O3	30	1,6	0
therapeutic ball O4	0,3	/	0,1
work desk O5	30	/	/
**INSTITUTION D**			

Physiotherapy			
therapeutic pillow P1	30	30	0,1
stationary bike P2	0,1	30	0
therapeutic bars P3	0	0	0,1
Stilts P4	0,2	30	30
stimulation electrodes P5	0,2	30	/
Occupational therapy			
plastic accessorise O1	0,1	30	0,3
wooden accessorise O2	30	30	0
hand bike O3	30	30	0,1
therapeutic ball O4	30	30	30
work desk O5	0,5	30	30
Common area			
Keyboard C1	/	17	0,1
coffee machine C2	/	20	25

Legend: P- physiotherapy; O – occupational therapy; C – common area; the number represents the sampled device.
